# The External Validation of a Multivariable Prediction Model for Recurrent Pelvic Organ Prolapse After Native Tissue Repair: A Prospective Cohort Study

**DOI:** 10.3390/jcm14020531

**Published:** 2025-01-15

**Authors:** Imke Kessels, Sander van Kuijk, Tineke Vergeldt, Iris van Gestel, Wilbert Spaans, Kim Notten, Roy Kruitwagen, Mirjam Weemhoff

**Affiliations:** 1 Department of Obstetrics and Gynecology, Zuyderland Medical Center, Henri Dunantstraat 5, 6419 PC Heerlen, The Netherlands; 2 Department of Obstetrics and Gynecology, Maastricht University Medical Center (MUMC+), P. Debyelaan 25, Postbus 5800, 6202 AZ Maastricht, The Netherlands; 3 Department of Clinical Epidemiology and Medical Technology Assessment (KEMTA), Maastricht University Medical Center (MUMC+), P. Debyelaan 25, Postbus 5800, 6202 AZ Maastricht, The Netherlands; 4 Department of Obstetrics and Gynecology, Gateshead Health NHS Foundation Trust, Sheriff Hill, Gateshead NE9 6SX, Tyne and Wear, UK; 5 Department of Obstetrics and Gynecology, Viecuri Medical Center, Tegelseweg 210, 5912 BL Venlo, The Netherlands; 6 Department of Obstetrics and Gynecology, Radboud University Medical Center, Geert Grooteplein Zuid 10, 6525 GA Nijmegen, The Netherlands

**Keywords:** external validation, native tissue repair, pelvic organ prolapse, prediction model, recurrence, risk factors, surgery

## Abstract

**Background/Objectives**: A prediction model for anatomical cystocele recurrence after native tissue repair was developed and internally validated in 2016. This model estimates a patients’ individual risk of recurrence and can be used for counseling. Before implementation in urogynecological clinical practice, external validation is needed. The aim of this study was to assess the external validity of this previously developed prediction model. The secondary aim was to test the performance of this model with a composite and subjective outcome of pelvic organ prolapse (POP) recurrence. Furthermore, the aim was to investigate whether risk factors for POP recurrence were in line with the population in which the original model was developed. **Methods**: In this prospective multicenter cohort study, 246 patients who underwent anterior colporrhaphy were included. Inclusion criteria were patients scheduled to undergo a primary anterior colporrhaphy (with a POP Quantification (POPQ) stage ≥ 2 cystocele). A combination of a primary anterior colporrhaphy with other POP or incontinence surgery (without the use of vaginal or abdominal mesh material) was permitted. Patients with prolapse or incontinence surgery prior to index surgery could not participate. All patients filled in questionnaires, pelvic floor ultrasound was performed preoperatively, and data from the medical file concerning POPQ stage and obstetric and general history were obtained. **Results**: Thirty women (12.2%) were lost at follow up. Anatomical cystocele recurrence was present in 107/216 (49.5%), subjective recurrence in 19/208 (9.1%), and 39/219 (17.8%) patients met the criteria for composite outcome. The area under the receiver operating characteristic curves for anatomical, composite, and subjective recurrence were 65.5% (95% CI: 58.7–72.4), 55.8% (95% CI 47.3–64.3%, NS), and 55.1% (95% CI 45.1–65.2%), respectively. In the multivariable analysis, preoperative cystocele stage 3 or 4 and a complete levator defect on ultrasound were independent risk factors for anatomical recurrence. For composite recurrence, younger age and an active employment status were only risk factors in univariable analysis. No significant risk factors for subjective recurrence could be identified. **Conclusions**: This external validation study showed a moderate performance for a prediction model for anatomical recurrence. The model cannot be used for a composite or subjective outcome prediction because of poor performance. For composite and subjective recurrence, new prediction models need to be developed.

## 1. Introduction

One of the most challenging aspects of urogynecological surgical practice is the risk of recurrence after native tissue repair. The anatomical risk of recurrence after anterior colporrhaphy is estimated to range up to 50% [1,2]. Native tissue repair is known to have high reoperation rates. However, individual recurrence risk may vary between patients. Identifying the individual risk of pelvic organ prolapse (POP) recurrence after surgery could be useful for counseling as it may influence treatment choices and can manage patients’ expectations.

Known risk factors for anatomical POP recurrence are a high preoperative stage (Pelvic Organ Prolapse Quantification (POPQ) stage 3 or 4) and a younger age [3]. In 2016, a prediction model was developed to estimate a patients’ individual risk of anatomical recurrence after anterior colporrhaphy, including assisted vaginal delivery, preoperative cystocele POPQ stage 3 or 4, number of compartments involved, a major levator defect on ultrasound, and the hiatal area during Valsalva as predictors [2]. The model was developed and internally validated in a sample of 287 women from two cohort studies. The area under the receiver operating characteristic (ROC) curve (AUC) was 69.7%, and the model showed good calibration.

Clinical prediction models need prospective, external validation before implementation in medical practice.

In the Netherlands, there is an increasing preference for uterine sparing surgery in the treatment of POP since recent studies indicate fewer anatomical recurrences, less repeat surgery, and a higher proportion of composite success when performing sacrospinous hysteropexy [4].

The aim of this study was to perform an external validation of the previously developed prediction model. Furthermore, we investigated the performance of the model for a composite outcome and evaluated whether the risk factors for recurrence were in line with the population in which the original model was developed.

## 2. Materials and Methods

This study is an observational prospective multicenter cohort study, with a one year follow-up of women undergoing primary anterior colporrhaphy in one of four hospitals (two university hospitals, two teaching hospitals). All participating hospitals also recruited women for the previous two cohort studies that were combined to develop the original prediction model [5,6]. This means that this study presents a temporal validation, where all characteristics remain the same except for the period of data collection. The secondary aims of the study were to investigate the performance of the model for a composite and a subjective outcome (and not only for anatomical recurrence) and to evaluate whether the risk factors for POP recurrence were in line with the population in which the original model was developed.

### 2.1. Inclusion and Exclusion Criteria

In the participating hospitals, all patients who were planned for an anterior colporrhaphy (or in combination with further POP surgery) with at least a cystocele POPQ of stage 2 were counseled for participation in this study. Patients with a history of POP surgery or incontinence surgery were excluded, as well as patients in which concomitant surgery with mesh material or implants (vaginal or abdominal mesh) was performed. Only participants who gave written informed consent could participate.

### 2.2. Study Protocol

Preoperatively and one year postoperatively, POP was staged using the POPQ system [7]. The POPQ staging was performed in standard 45-degrees supine position. The postoperative POPQ staging was performed by another physician than the surgeon, to prevent bias.

Patients were asked to fill in a questionnaire prior to surgery and one year after surgery. The preoperative questionnaire consisted of questions on baseline characteristics, e.g., obstetrical history, education, current profession, and active employment status. Disease-specific quality of life regarding the presence and severity of urogynecological symptoms was measured using the Urogenital Distress Inventory (UDI) [8].

Patients underwent 3D translabial ultrasound of the pelvic floor preoperatively. Three-dimensional translabial ultrasound was performed after voiding in supine position with the hips flexed and slightly abducted. A 3D ultrasound machine (Voluson GE Kretz Ultrasound, Zipf, Austria) and a 4–8 Mhz curved abdominal transducer were utilized to perform the ultrasound. The transducer was placed against the perineum in the mid-sagittal plane with a maximum angle of 70 degrees for 2D imaging. The dimensions of the levator hiatus were determined in the oblique axial plane at the level of minimum hiatal dimensions, identified as the minimum distance between the posterior margin of the symphysis pubis and the anterior margin of the puborectal muscle. In this plane, a 4D volume cine was recorded during contraction and during Valsalva.

The levator hiatal area during Valsalva was measured in square centimeters as the area bordered by the levator ani muscle, the symphysis pubis, and the inferior pubic ramus.

Tomographic ultrasound imaging was used to assess levator ani muscle defects. Levator damage was graded by the classification system developed by Dietz et al. in the three central slices (i.e., the plane of minimal dimensions plus slices 2.5 mm and 5 mm above this plane) [9]. The recorded data were stored anonymously (using the study number) and were analyzed offline by two gynecologists who were blinded to the associated clinical data. The patient and the operating gynecologist were blinded to the ultrasound measurements.

### 2.3. Definitions

Anatomical recurrence in this study was defined as POP in the anterior compartment equal to or greater than POPQ stage 2 and/or retreatment of the anterior compartment (surgery or pessary) in the first year after surgery. Subjective recurrence was defined as feeling and/or seeing a vaginal bulge, with at least a judgment of one of these two symptoms as moderately bothersome or both of these symptoms as somewhat bothersome, according to the UDI which was taken one year after surgery. Composite recurrence was defined as a POP beyond the hymen in any compartment, subjective recurrence, and/or retreatment (surgery or pessary) because of recurrent POP in the first year after surgery.

### 2.4. Statistical Analysis

The sample size was calculated on the primary objective of this study, i.e., external validation of a previously published prediction model. Collins et al. [10] recommended that externally validating a prognostic model requires a minimum of 100 events and 100 non-events. Since the anatomical risk of recurrence after primary colporrhaphy was estimated at 50% or more, data from 200 women were needed. We decided on a total sample size of 260 patients to account for loss to follow-up. The women of the development cohort were compared with the women of this external validation cohort by using the independent samples t-test for continuous variables, and Pearson’s chi-square test for categorical variables.

Missing predictor values necessary to compute an individual’s prediction were imputed using stochastic regression imputation with fully conditional specification and predictive mean matching, as using only complete cases may cause a severe loss of statistical precision. To determine the performance of the prediction model, each individual’s risk of anatomical recurrence was calculated using the formula derived from the published manuscript. Their predicted risk of recurrence and each individual’s outcome was used to compute the parameters of model performance. The discriminative ability was quantified as the AUC.

The model calibration for the anatomical recurrence was determined in two steps. First, we computed the calibration-in-the-large, which is the difference between the average predicted risk of recurrence in the whole cohort and the observed frequency of the outcome. It indicates whether the model systematically over- or underestimates the predicted risks. The prediction model was recalibrated in case the calibration-in-the-large substantially deviated from zero, by re-estimating the intercept and slope using the linear predictor as the only covariate in a regression model. Subsequently, we made a calibration plot of the average predicted risk of recurrence and the observed frequency for subgroups based on similar risks. All significant deviations from the ideal 45-degree line of equality are evidence of poor calibration.

For the secondary aim, women with and without recurrence were compared with respect to age, body mass index (BMI), assisted vaginal delivery, preoperative cystocele POPQ stage 3 or 4, preoperative POPQ stage 3 or 4 in any compartment, number of compartments involved, concomitant POP surgery, levator ani defects on 3D ultrasound during contraction, levator hiatal area during Valsalva on 3D ultrasound, being sexually active, and an active employment status.

Multivariable logistic regression, including all factors with *p* < 0.10 in the univariable analysis, was used to select independent predictors of recurrence. Results were expressed as odds ratios (ORs) and 95% confidence intervals (95% CIs). A *p* < 0.05 was considered statistically significant, and all statistical analyses were performed using SPSS version 28 and R version 4.0.2.

The TRIPOD (Transparent Reporting of a multivariable prediction model for Individual Prognosis or Diagnosis) guidelines were used when composing this manuscript [11].

### 2.5. Ethical Approval

This study was approved by the Medical Ethics Committee of the Maastricht University Medical Center (MUMC+). Ethical approval was obtained on 1st of May 2017. Local approval was obtained in all participating centers. The trial is registered in the Dutch Trial Register (NTR, Nederlands trial register) with registration number NL6292, Registered 25 May 2017, https://onderzoekmetmensen.nl/nl/trial/55841 (accessed on 1 December 2024).

## 3. Results

In this study, 260 patients were enrolled between July 2017 and October 2021. The baseline characteristics are shown in Table 1. Appendix A shows the patient flow of the study, and fourteen patients were excluded after initial inclusion. Of the 246 women remaining in the cohort, 216 women (87.8%) attended the follow-up visit for POPQ staging one year postoperatively and 208 (84.6%) women completed the postoperative questionnaire. There were no clinically relevant differences between patients who did and did not attend the follow-up visit.

From 181 women (73.6%), ultrasound data were available to assess for levator defects, and from 171 women (69.5%), data were available to measure the hiatal area during Valsalva. The mean period between surgery and follow up was one year and two months (range of 283–1086 days); this was slightly longer than one year because follow-up appointments were delayed due to the COVID19 pandemic.

There were significant differences in baseline characteristics between the developmental cohort and this external validation cohort (Table 1). In the external validation cohort, patients were slightly older (61.1 versus 58.0 years, *p* < 0.001), were less frequently operated on in only one compartment (8.1% vs. 24.7%, *p* < 0.001) and more frequently in two compartments (61.4% vs. 44.6% *p* < 0.001), and the number of patients with a POPQ stage 3 or 4 was lower (43.1% vs. 53.1%, *p* 0.02. In the external validation cohort, the number of surgeries on the posterior compartment was lower (35.8% vs. 44.6% *p* 0.04), but the number of surgeries on the middle compartment was higher (78.9% vs. 61.0% *p* < 0.001), with more uterine sparing surgery. The ultrasound characteristics also showed significant differences: in the external validation cohort, the mean levator hiatal area was smaller (28.9 vs. 33.3 cm^2^ *p* < 0.001), and there were fewer patients with complete levator defects (28.6% vs. 45.2%).

In the external validation cohort, 107/216 patients (49.5%) had anatomical recurrence, compared to 151/287 patients (52.6%) in the developmental cohort (*p* = 0.47).

In the external validation cohort, 19/208 patients (9.1%) met the criteria for subjective recurrence compared to 28/283 patients (9.9%) in the developmental cohort (*p* = 0.77).

In 39/219 patients (17.8%) in the external validation cohort, the criteria for composite recurrence were met. In the developmental cohort, the composite outcome could not be assessed because only the POPQ stages had been collected instead of individual POPQ points.

There were seven patients who received a new treatment for recurrent POP. Five patients received a pessary, and two patients were re-operated in the first year after the index surgery. Twenty-eight out of 129 patients (21.7%) who underwent an SSF had an anatomical recurrence.

**Figure 1:**
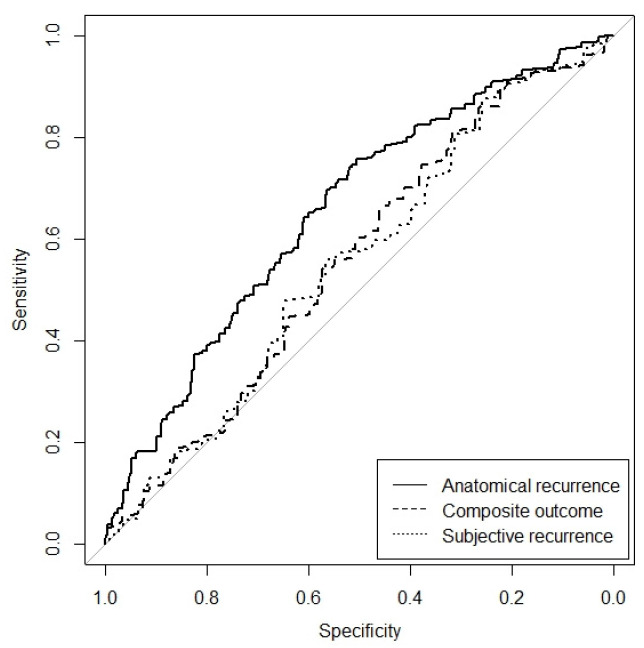
Receiver Operating Curves (ROC) for the external validation of the prediction for anatomical recurrence and composite recurrence. AUC 65.5% (95% CI: 58.7–72.4%) for anatomical recurrence (straight line) and 55.8% (95% CI: 47.3–64.3%, NS) for composite recurrence (striped line) and 55.1% (95% CI 45.1–65.2%), for subjective recurrence (dotted line).

**Figure 2:**
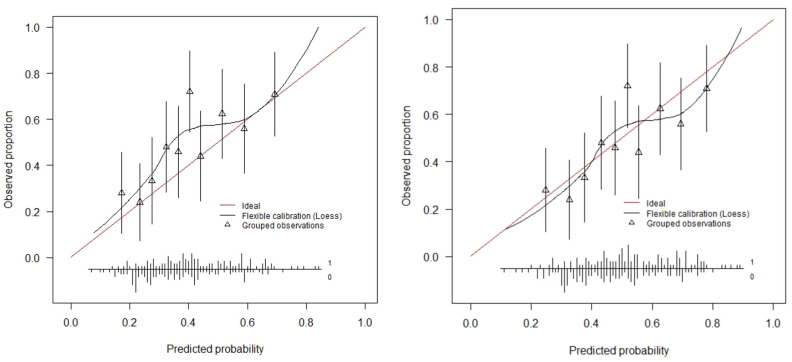
Calibration plot with the original intercept (−2.35) on the left, showing the predicted probabilities of anatomical recurrence for grouped patients based on similar predicted risk compared with the observed frequency of events per group. There is slight underestimation of the actual probability. On the right the calibration plot with the adjusted intercept (−1.89).

The AUC for anatomical recurrence was 65.5% (95% CI: 58.7–72.4%) in this external validation cohort, which expresses the discriminative ability of this model (Figure 1). The average predicted risk of an anatomical recurrence according to the prediction model was 40.2% (range 8–84%). The actual anatomical recurrence rate was 49.5%. This means that, on average, the model underestimated recurrence risks since there is a difference between the observed risk and the predicted risk (i.e., the calibration-in-the-large is moderate). The calibration plot depicts this systematic underestimation (Figure 2). For perfectly calibrated models, we should observe a straight 45 degree line, so that the estimated risks are always the same as the real ones.

In Figure 2, we see that the predicted risks are lower than the observed risks. The intercept of the prediction model, which was −2.35 in the original model, was subsequently re-estimated on all available data to arrive at a more precise estimate. With the new intercept of −1.89, the average predicted risk was calculated as 47.6%. (Figure 2).

The adjusted prediction model, with the adjusted intercept (−1.89) is shown in Box 1, the original prediction model is shown in Box 2.

Box 1 **Adjusted prediction model.**

P (recurrence) = 1/(1 + exp(−(Linear predictor))) in which linear predictor = −1.89 − 0.57 × assisted vaginal delivery (yes = 1) + 0.82 × preoperative stage 3 or 4 (yes = 1) − 0.25 × number of compartments (1, 2 or 3) + 0.28 × major levator defect (yes = 1) + 0.07 × levator hiatal area (in cm^2^).

Box 2 **Original prediction model after development and internal validation.**

P (recurrence) = 1/(1 + exp(−(Linear predictor))) in which linear predictor = −2.35 − 0.57 × assisted vaginal delivery (yes = 1) + 0.82 × preoperative stage 3 or 4 (yes = 1) − 0.25 × number of compartments (1, 2 or 3) + 0.28 × major levator defect (yes = 1) + 0.07 × levator hiatal area (in cm^2^).

This adjusted formula for the anatomical prediction model was consequently used for calculating the performance of the model for composite and subjective outcome to evaluate whether it would be applicable for a composite outcome. The AUC of the ROC curve was 55.8% (95% CI 47.3–64.3%, NS) for composite recurrence and 55.1% (95% CI 45.1–65.2%) for subjective recurrence, showing poor performance for both outcomes (Figure 1).

Table 2 shows an overview of all the assessed risk factors for anatomical, composite, and subjective recurrence in our study population. Risk factors in the univariable analysis for anatomical recurrence were preoperative cystocele POPQ stage 3 or 4, POPQ stage 3 or 4 in any compartment preoperatively, concomitant SSF, and a complete levator defect. In multivariable logistic regression analysis, as shown in Table 3, only preoperative cystocele POPQ stage 3 or 4 and a complete levator defect turned out to be independently associated with anatomical recurrence.

In the univariable logistic regression analysis, younger age and an active employment status appeared to be a risk factors for composite recurrence, but were non-significant in the multivariable analysis. No significant risk factors were identified for subjective recurrence.

## 4. Discussion

This study assesses the external validation of a prediction model for anatomical recurrence after anterior colporrhaphy. The external validation of prediction models is highly important because models tend to perform too optimistically in the developmental sample [12]. Our study confirms this statement, although the AUC decreased only slightly.

When comparing the developmental and the external validation cohort, the anatomical recurrence rates were similar. However, there were significant differences in patient characteristics between the cohorts, with less patients operated on in one compartment only and more surgery in the middle compartment in the external validation cohort.

Furthermore, in the external validation cohort, more uterine sparing surgery was performed. This shift towards more uterine sparing surgery reflects the change in treatment preferences in the Netherlands because of recent studies on the results of uterine sparing surgery [4]. The populations studied in both the developing and the validation cohort are representative of the general pelvic organ prolapse population as they were treated in the Netherlands. Even in a population with different patient characteristics, the discriminative performance of the model was only slightly lower, showing the transportability of the model.

A strength of this study is that this external validation was performed in a sample from the same hospitals as for the development. There were no conversions or redefinitions of variables due to differences in measurements or definitions. Thereby, a potential difference in performance of the model would not result from non-identified heterogenic differences between the developmental and validation cohorts. A weakness of this study is the proportion of missing values. An important cause of missing values was ultrasound files with technical failures or volumes, lacking the adequate reference points for assessment. This might have influenced the results because, especially in cases with a large hiatal area, it can be difficult to visualize these reference points.

Furthermore, extending the follow-up period could help to gain deeper insights into the long-term risk factors for prolapse recurrence.

In this study, we found a complete levator defect and a high preoperative cystocele stage to be independently associated with anatomical recurrence. These risk factors correspond with the observed risk factors in the developmental cohort. In the study by Weemhoff et al. [5], a complete levator defect, SSF, a positive family history, and a higher preoperative POP stage were found to be independent risk factors for anatomical recurrence.

Furthermore, in the study of Friedman et al. [13], a high preoperative stage and larger hiatal area were associated with recurrence. We could not confirm the larger hiatal area being a risk factor, but a hiatal area <20 cm^2^ was a protective factor for anatomical recurrence in the univariable analysis.

In recent years, there has been considerable debate on the ideal outcome measure of treatment success or failure after POP surgery [14]. Despite the lack of an internationally accepted definition, there is nowadays a consensus that the absence of vaginal bulge symptoms should be included in the definition of composite success, since this absence has a strong relationship with patients’ assessment of overall improvement [15]. The present model unfortunately cannot be used for a composite outcome as the AUC for composite recurrence was only 55.8%, reflecting poor discrimination.

Very few studies report on risk factors for recurrent POP with composite or subjective outcome measures after native tissue repair [16,17]. In our study, the risk factors for anatomical recurrence appeared to not be significant for the composite outcome, as only a younger age was associated with composite recurrence. In a recent review on risk factors for recurrence, it was found that a younger age was a risk factor for anatomical recurrence [3]. A possible explanation for this finding could be that younger patients experience more bother from recurrent POP than older patients.

The need for objective risk assessment tools is increasing, since clinicians want to provide individualized information when counseling their patients on treatment options. For counseling on POP recurrence, the availability of prediction models is limited. One study reported on the development and internal validation of multivariable prediction models for POP recurrence [18], but these models have never been externally validated. This is a common problem in prediction models: only 9% of all prediction models in the field of benign gynecology are externally validated [19]. Our study shows a robust external validation conducted in a prospective, multicenter setting.

In urogynecological clinical practice and research, we agree upon the importance of composite, subjective, and satisfaction outcomes. However, we should not undervalue the knowledge we have gained on risk factors for anatomical recurrence and predicting anatomical recurrence as long as this information is lacking for the other outcome measures. We should possibly focus on studying a different type of risk factor for subjective and satisfaction outcomes, such as body image or anxiety scores. The goal for future research is to continue developing a prediction model for composite recurrence.

## Figures and Tables

**Table 1:** Patient characteristics of the developmental and validation cohort.

	Developmental Cohort (*n* = 287) N (%) or Mean (±SD)	Validation Cohort (*n* = 246) N (%) or Mean (±SD)	*p*-Value
Age, years	58.0 (±10.3)	61.1 (±9.1)	<0.001
BMI, kg/m^2^	26.0 (±3.9)	26.0 (±3.7)	0.89
Assisted vaginal delivery	32/276 (11.6%)	13.5%	0.51
POPQ stage 3 or 4 cystocele preoperatively	132/285 (46.3%)	94/246 (38.2%)	0.06
POPQ stage 3 or 4 in any compartment preoperatively	152/286 (53.1%)	106/246 (43.1%)	0.02
Complete levator defect	126/279 (45.2%)	58/203(28.6%)	<0.001
Levator hiatal area (cm^2^)	33.3 (±8.0)	28.9 (±7.1)	<0.001
Levator ballooning			
Hiatal area < 20 cm^2^	11/271 (4.1%)	17/193 (8.8%)	0.03
Hiatal area > 25 cm^2^	237/271(87.5%)	131/193 (67.9%)	<0.001
Number of compartments involved			
Anterior colporrhaphy only	71/287 (24.7%)	20/246 (8.1%)	<0.001
Surgery in 2 compartments	128/287 (44.6%)	151/246 (61.4%)	<0.001
Surgery in anterior and posterior compartment	40/287 (13.9%)	13/246 (5.3%)	<0.001
Surgery in anterior and middle compartment	88/287 (30.7%)	138/246 (56.1%)	<0.001
Surgery in 3 compartments	88/287 (30.7%)	75/246 (30.5%)	0.97
Concomitant repair of uterus or vaginal vault			
Vaginal hysterectomy	102/287 (35.5%)	19/246 (7.7%)	<0.001
Sacrospinous fixation	46/287 (16.0%)	129/246 (52.4%)	<0.001
Manchester Fothergill	27/287 (9.4%)	65/246 (26.4%)	<0.001

BMI: body mass index. POPQ: Pelvic Organ Prolapse Quantification. OR: Odds Ratio. CI: Confidence Interval.

**Table 2:** Univariable analysis for anatomical, composite and subjective recurrence.

	Anatomical Recurrence			Composite Recurrence			Subjective Recurrence		
	*n*	OR (95%CI)	*p*-Value	*n*	OR (95%CI)	*p*-Value	*n*	OR (95%CI)	*p*-Value
Age, years	216	1.02 (0.99–1.05)	0.22	219	0.96 (0.92–0.99)	0.04	208	0.96 (0.91–1.01)	0.13
Younger age (<60 years)	216	0.86 (0.50–1.47)	0.58	219	1.56 (0.78–3.13)	0.21	208	1.10 (0.43–2.83)	0.84
BMI kg/m^2^	216	1.03 (0.96–1.10)	0.5	219	1.04 (0.95–1.13)	0.38	208	1.07 (0.96–1.21)	0.23
BMI > 25	216	1.11 (0.66–1.88)	0.69	219	1.05 (0.54–2.05)	0.89		1.23 (0.50–3.25)	0.62
BMI > 30	216	0.79 (0.36–1.77)	0.57	219	0.89 (0.32–2.47)	0.82		1.95 (0.61–6.28)	0.26
Assisted vaginal delivery	209	0.46 (0.20–1.07)	0.07	212	0.98 (0.35–2.77)	0.97	204	1.39 (0.37–5.15)	0.62
POPQ stage 3 or 4 cystocele preoperatively	216	2.42 (1.40–4.28)	0.002	219	1.44 (0.71–2.90)	0.31	208	0.61 (0.21–1.76)	0.36
POPQ stage 3 or 4 in any compartment preoperatively	216	1.70 (0.98–2.93)	0.06	219	1.39 (0.70–2.79)	0.35	208	0.80 (0.30–2.11)	0.65
POPQ Genital hiatus (on Valsalva)	173	1.03 (0.81–1.30)	0.84	176	0.93 (0.67–1.29)	0.65	165	0.59 (0.33–1.06)	0.08
Levator defect	181		0.007	183		0.75	176		0.65
No levator defect (ref)									
Partial levator defect		1.60 (0.68–3.76)	0.28		0.83 (0.36–2.70)	0.76		0.38 (0.05–3.11)	0.37
Complete levator defect		3.06 (1.52–6.18)	0.002		1.28 (0.56–2.96)	0.56		1.01 (0.33–3.13)	0.99
Levator hiatal area (cm^2^)	171	1.03 (0.99–1.08)	0.15	173	1.01 (0.95–1.07)	0.79			
Hiatal area < 20 cm^2^		0.35 (0.10–1.13)	0.08		1.02 (0.27–3.80)	0.98	166	0.79 (0.09–5.72)	0.74
Hiatal area > 25 cm^2^		1.40 (0.73–2.68)	0.32		1.20 (0.51–2.80)	0.68		3.22 (0.70–14.81)	0.13
Number of compartments involved	216		0.33	219		0.63	208		0.68
Anterior colporrhaphy only (ref)									
Surgery in 2 compartments		2.19 (0.78–6.20)	0.14		1.94 (0.42–8.99)	0.4		1.89 (0.23–15.45)	0.55
Surgery in 3 compartments		1.89 (0.63–5.61)	0.25		1.54 (0.31–7.79)	0.6		1.27 (0.14–11.65)	0.83
Posterior colporrhaphy	216	0.88 (0.50–1.53)	0.64	219	0.88 (0.46–1.94)	0.94	208	0.95 (0.36–2.52)	0.92
Repair of uterus or vaginal vault									
Vaginal hysterectomy	216	1.02 (0.37–2.82)	0.97	219	0.64 (0.14–2.94)	0.57	208	1.47 (0.31–7.02)	0.63
Sacrospinous fixation	216	1.89 (1.10–3.24)	0.02	219	1.19 (0.60–2.39)	0.62	208	0.44 (0.16–1.20)	0.11
Manchester Fothergill	216	0.65 (0.36–1.18)	0.16	219	0.99 (0.46–2.14)	0.99	208	1.87 (0.71–4.90)	0.21
Active employment status	200	0.82 (0.47–1.43)	0.48	203	2.25 (1.04–4.86)	0.04	195	2.46 (0.84–7.18)	0.10
Sexually active	207	0.88 (0.48–1.60)	0.68	210	1.06 (0.47–2.36)	0.89	202	3.43 (0.76–15.47)	0.11

BMI: body mass index. POPQ: Pelvic Organ Prolapse Quantification. OR: Odds Ratio. CI: Confidence Interval.

**Table 3:** Multivariable analysis for anatomical and composite recurrence.

	Anatomical Recurrence			Composite Recurrence		
	*n*	OR (95%CI)	*p*-Value	*n*	OR (95%CI)	*p*-Value
Age				219	0.99 (0.94–1.05)	0.88
Assisted vaginal delivery	209	0.59 (0.22–1.61)	0.31			
POPQ stage 3 or 4 cystocele preoperatively	216	2.33 (1.18–4.58)	0.02			
Levator defect No levator defect (ref) Partial levator defect Complete levator defect	181	2.98 (1.40–6.43)	0.02 0.01			
Levator hiatal area (cm^2^) Hiatal area < 20 cm^2^ Hiatal area >25 cm^2^	171	0.41 (0.11–1.48)	0.17			
Repair of uterus or vaginal vault Vaginal hysterectomy Sacrospinous fixation Manchester Fothergill	216	1.69 (0.87–3.30)	0.12			
Active employment status				203	2.14 (0.78–5.88)	0.14

POPQ: Pelvic Organ Prolapse Quantification. OR: Odds Ratio. CI: Confidence Interval.

## Data Availability

The raw data supporting the conclusions of this article will be made available by the authors on request.

## Appendix A

Patient Flow Chart
